# Prognostic Impact of Baseline Albumin–Bilirubin Score on Mortality After Transcatheter Edge-to-Edge Mitral Repair

**DOI:** 10.3390/medicina62050944

**Published:** 2026-05-12

**Authors:** Ümeyir Savur, Berhan Keskin, Aysel Akhundova, Aykun Hakgor, Haci Murat Güneş, Bilal Boztosun

**Affiliations:** Department of Cardiology, Istanbul Medipol University, Medipol Mega University Hospital, 34214 Istanbul, Turkey; berhankeskin92@gmail.com (B.K.); aysel.akhundova@yahoo.com (A.A.); aykunhakgor@gmail.com (A.H.); dr_muratgunes@hotmail.com (H.M.G.); bilal.boztosun@medipol.edu.tr (B.B.)

**Keywords:** mitral regurgitation, transcatheter edge-to-edge repair, ALBI score, cardio-hepatic interaction, risk stratification

## Abstract

*Background and Objectives*: Transcatheter edge-to-edge repair (TEER) has emerged as an effective treatment option for patients with severe mitral regurgitation who are at high surgical risk. However, clinical outcomes after TEER remain heterogeneous and are influenced not only by cardiac parameters but also by systemic comorbidities and multiorgan dysfunction. The albumin–bilirubin (ALBI) score, derived from serum albumin and bilirubin levels, has recently been proposed as a simple marker of hepatic dysfunction and cardio-hepatic interaction. This study aimed to evaluate the prognostic value of baseline ALBI score in predicting long-term mortality after TEER. *Materials and Methods*: In this single-center retrospective cohort study, 106 consecutive patients with symptomatic moderate-to-severe or severe mitral regurgitation who underwent TEER between January 2019 and December 2025 were included. Baseline ALBI score was calculated using pre-procedural serum albumin and bilirubin levels. Cox proportional hazards regression analysis was used to identify predictors of long-term mortality. Variable selection was performed using least absolute shrinkage and selection operator (LASSO) regression, followed by ridge-penalized multivariable Cox modeling to minimize overfitting. The incremental prognostic value of ALBI was assessed using concordance index (C-index) comparison between predictive models. Receiver operating characteristic (ROC) analysis and Kaplan–Meier survival analysis were also performed. *Results*: During a median follow-up of 17.9 months, 30 patients (28.3%) died. Higher baseline ALBI scores were significantly associated with increased mortality risk. In multivariable analysis, ALBI score (HR 3.35, 95% CI 1.46–7.71; *p* = 0.004), left atrial volume index (LAVI) (HR 1.02, 95% CI 1.01–1.03; *p* = 0.005), and log-transformed B-type natriuretic peptide (BNP) (HR 1.37, 95% CI 1.02–1.86; *p* = 0.039) remained independent predictors of mortality. Addition of the ALBI score improved model discrimination, increasing the C-index from 0.845 to 0.886. ROC analysis demonstrated good predictive performance of the ALBI score (area under the curve [AUC] = 0.877), with an optimal cut-off value of −1.67. *Conclusions*: Baseline ALBI score is independently associated with long-term mortality after TEER and may provide potential incremental prognostic information. However, the observed improvement is modest and should be interpreted cautiously. These findings support a potential role of ALBI as a complementary marker, which requires validation in larger prospective studies.

## 1. Introduction

Severe mitral regurgitation (MR) is a progressive valvular disorder with increasing prevalence in aging populations and remains associated with substantial morbidity and mortality despite advances in contemporary heart failure (HF) therapy [1]. Chronic volume overload contributes to progressive left ventricular remodeling, worsening HF symptoms, pulmonary hypertension, and ultimately systemic end-organ dysfunction [1]. In patients with high or prohibitive surgical risk, transcatheter edge-to-edge repair (TEER) using the MitraClip™ system (Abbott Vascular, Santa Clara, CA, USA) has emerged as an effective alternative to surgery, offering durable MR reduction and clinically meaningful symptomatic improvement across a broad spectrum of HF phenotypes [2,3]. Nevertheless, despite high procedural success rates, post-TEER outcomes remain heterogeneous and appear to be strongly influenced by baseline patient characteristics, including ventricular function, pulmonary pressures, renal dysfunction, systemic inflammatory burden, and the extent of venous congestion and hepatic impairment [4].

Chronic systemic venous congestion is a central feature of advanced HF and an important hemodynamic driver of multiorgan injury. Sustained elevation in right-sided filling pressures may lead to hepatic abnormalities collectively termed cardiac hepatopathy, characterized by impaired bilirubin clearance, reduced albumin synthesis, and progressive congestion-related liver dysfunction [4,5]. Conventional hepatic scoring systems, such as Child–Pugh and Model for End-Stage Liver Disease (MELD), were not originally designed to assess cardiogenic liver injury and may therefore have limited discriminatory value in patients with HF-related congestion [5].

The albumin–bilirubin (ALBI) score, initially developed to objectively assess hepatic dysfunction in patients with hepatocellular carcinoma using only serum albumin and total bilirubin levels [6], has increasingly attracted attention in cardiovascular research. Because it is derived exclusively from routinely available laboratory parameters and does not rely on subjective clinical components, ALBI provides a simple and objective estimate of hepatic functional reserve. Recent evidence suggests that ALBI may reflect congestion-related hepatic dysfunction and global systemic vulnerability in patients with structural heart disease, including those undergoing transcatheter aortic valve implantation and transcatheter tricuspid interventions [7]. In the HF setting, ALBI may therefore represent an integrated biomarker of the cardio-hepatic interaction, capturing the downstream effects of chronic venous congestion beyond conventional cardiac risk indicators.

However, the prognostic significance of baseline ALBI score in patients undergoing TEER has not yet been fully elucidated. In particular, it remains uncertain whether pre-procedural hepatic dysfunction independently contributes to mortality risk after TEER and whether ALBI offers incremental prognostic value beyond established clinical and echocardiographic predictors [8]. Identifying patients at increased risk of adverse outcomes is clinically relevant for optimizing procedural timing, refining peri-procedural management, and supporting individualized therapeutic decision making in this high-risk population.

Long-term outcomes after TEER have been extensively investigated, and long-term mortality remains a clinically meaningful endpoint, as it may reflect the cumulative burden of systemic and end-organ dysfunction present at the time of intervention. Therefore, the present study aimed to evaluate whether baseline ALBI score predicts long-term all-cause mortality after TEER and whether it provides incremental prognostic information beyond established clinical and echocardiographic risk markers.

## 2. Materials and Methods

### 2.1. Study Design and Population

This single-center retrospective cohort study included consecutive patients with symptomatic moderate-to-severe (3+) or severe MR (4+) who underwent TEER using the MitraClip™ system (Abbott Vascular, Santa Clara, CA, USA) between January 2019 and December 2025.

All patients were considered to be at high or prohibitive surgical risk following comprehensive evaluation by the institutional multidisciplinary Heart Team, consisting of interventional cardiologists, cardiac surgeons, echocardiographers, anesthesiologists, and heart failure specialists. Eligibility for TEER was determined according to contemporary guideline recommendations and institutional procedural protocols [8].

**Figure 1:**
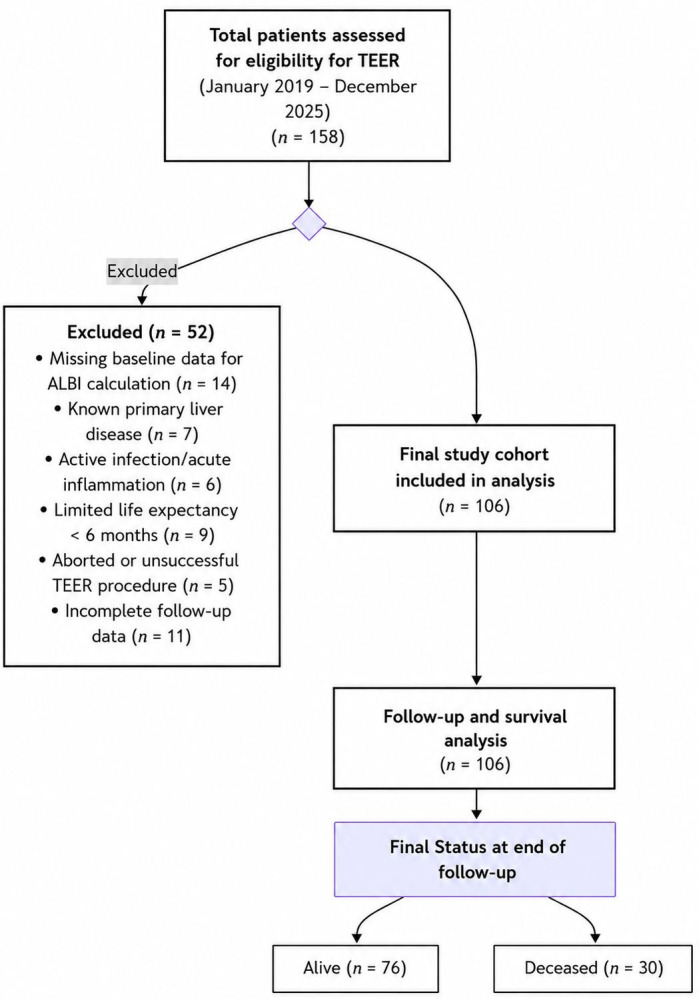
Flow diagram of patient selection.

Patient selection and exclusion criteria are summarized in a STROBE-compliant flow diagram (Figure 1).

A total of 158 consecutive patients evaluated for TEER due to symptomatic moderate-to-severe or severe MR between January 2019 and December 2025 were retrospectively screened. Given the retrospective design of the study, all data were collected and analyzed after completion of the study period. Ethical approval was obtained in January 2026 for retrospective data collection and analysis of previously treated patients. Patients were eligible for inclusion if they met both anatomical and clinical criteria for TEER and had complete baseline laboratory data required for the calculation of the ALBI score.

The exclusion criteria were as follows:

- Anticipated life expectancy <6 months due to non-cardiac comorbidities;
- Active infection or acute inflammatory conditions at the time of intervention;
- Known primary liver disease (e.g., cirrhosis, portal hypertension, or chronic viral hepatitis);
- Missing pre-procedural laboratory data;
- Aborted procedures or unsuccessful clip implantation.

After these exclusion criteria were applied, a final analytic cohort of 106 patients was identified and included in this study.

### 2.2. Clinical and Echocardiographic Assessment

Baseline demographic characteristics, comorbidities, and laboratory parameters were extracted from institutional electronic medical records. Pre-procedural transthoracic echocardiography (TTE) and transesophageal echocardiography (TEE) were systematically performed to assess mitral valve morphology, quantify MR severity, and determine anatomical eligibility for TEER [9,10]. Post-procedural TEE was performed immediately after the procedure in the operation room, and further evaluation with post-procedural TTE was done at the time of hospital discharge.

Mitral regurgitation severity was graded using an integrative, multiparametric approach incorporating qualitative, semi-quantitative, and quantitative parameters, including effective regurgitant orifice area and regurgitant volume. Transesophageal echocardiography was specifically utilized to evaluate leaflet anatomy, coaptation characteristics, and spatial relationships relevant to clip implantation. Procedural success was further categorized as optimal (≤1+ residual MR) or acceptable (2+ residual MR), in accordance with contemporary outcome reporting standards. Left ventricular ejection fraction (LVEF) was assessed using biplane Simpson’s method.

### 2.3. Device and Procedural Details

The MitraClip™ system (Abbott Vascular, Santa Clara, CA, USA) and the implantation technique have been described previously [11,12]. All procedures in the present study were performed according to standard institutional practice and in accordance with contemporary guideline recommendations.

Transcatheter edge-to-edge repair was performed under continuous transesophageal echocardiographic and fluoroscopic guidance. Vascular access was obtained via the femoral vein in all patients. Procedures were carried out under either general anesthesia with endotracheal intubation or deep sedation with spontaneous breathing, based on the assessment of the anesthesiology team.

After completion of the procedure, patients were monitored in the catheterization laboratory and subsequently transferred to the intensive care unit for post-procedural observation. Procedural success was defined as successful clip implantation with reduction in mitral regurgitation to ≤moderate at the end of the procedure without procedural mortality. Femoral venous hemostasis was achieved using a Z-suture technique followed by manual compression, and patients were maintained on bed rest for approximately 8 h.

### 2.4. Assessment of Hepatic Dysfunction and ALBI Calculation

Congestion-related hepatic dysfunction was assessed using the ALBI score, derived from serum albumin and total bilirubin levels measured within 24–48 h prior to the TEER procedure. The ALBI score provides an objective assessment of hepatic function using routinely available laboratory parameters and has been increasingly explored as a marker of cardio-hepatic interaction in patients with heart failure and structural heart disease.

The ALBI score was calculated using the following formula [6]:

ALBI = (0.66 × log10 [bilirubin (μmol/L)]) − (0.085 × albumin [g/L])

For bilirubin values reported in mg/dL, a conversion factor of 1 mg/dL = 17.1 μmol/L was applied. Based on previously established cut-off values [6], patients were categorized into three ALBI grades reflecting the severity of hepatic dysfunction: ALBI Grade 1 (≤−2.60), Grade 2 (−2.60 to −1.39), and Grade 3 (>−1.39). These grades were used for patient stratification and subsequent outcome analyses.

### 2.5. Clinical Endpoints

The primary endpoint of this study was long-term all-cause mortality following TEER.

### 2.6. Statistical Analysis

Continuous variables were assessed for normality and presented as mean ± standard deviation (SD) or median with interquartile range (IQR) as appropriate, while categorical variables were expressed as counts and percentages. Comparisons between groups were performed using the Student’s t-test or Mann–Whitney U test for continuous variables and the chi-square test or Fisher’s exact test for categorical variables.

Predictors of long-term mortality were evaluated using Cox proportional hazards regression analysis. Variables associated with mortality in univariate Cox analysis (*p* < 0.05) were entered into a least absolute shrinkage and selection operator (LASSO) Cox regression model for variable selection. Variables with non-zero coefficients were considered potential predictors and were included in the multivariable model.

Given the limited number of outcome events, a ridge-penalized Cox proportional hazards model was used for multivariable analysis to reduce the risk of model overfitting. Hazard ratios (HRs) with 95% confidence intervals (CIs) were reported. To assess model stability and potential optimism, internal validation was performed using bootstrap resampling with 500 iterations. In each bootstrap sample, the ridge-penalized Cox model was refitted, and model discrimination was evaluated both in the bootstrap sample and in the original dataset using Harrell’s concordance index. The optimism-corrected C-index was calculated by subtracting the mean optimism from the apparent C-index. To evaluate potential collinearity among variables included in the multivariable model, pairwise correlations were evaluated using Pearson correlation coefficients. A correlation coefficient ≥ 0.70 was considered indicative of strong correlation.

To assess the incremental prognostic value of the ALBI score, two models were compared. Model A included age, log-transformed B-type natriuretic peptide (BNP), and left atrial volume index (LAVI), while Model B additionally included the ALBI score. Model discrimination was evaluated using the concordance index (C-index).

To further evaluate the incremental prognostic value of the ALBI score, continuous net reclassification improvement (NRI) was calculated at 24 months by comparing two models. Model A included age, log-transformed BNP, and left atrial volume index (LAVI), while Model B additionally incorporated the ALBI score. Individual predicted risks at 24 months were derived from Cox regression models. Bootstrap resampling with 1000 iterations was performed to estimate 95% confidence intervals for the NRI.

Receiver operating characteristic (ROC) curve analysis was performed to evaluate the ability of the ALBI score to predict long-term mortality. The area under the curve (AUC) was calculated and the optimal cut-off value for the ALBI score was determined using Youden’s index.

Patients were subsequently stratified according to the identified ALBI cut-off value, and Kaplan–Meier survival curves were constructed to assess differences in long-term mortality. Survival between groups was compared using the log-rank test. To avoid unstable survival estimates caused by the small number of patients remaining at risk during longer follow-up, Kaplan–Meier curves were truncated at 24 months.

All statistical tests were two-sided, and a *p*-value < 0.05 was considered statistically significant. Statistical analyses were performed using Python version 3.14.0 (Python Software Foundation, Wilmington, DE, USA) within a dedicated virtual environment.

## 3. Results

The median follow-up duration was 17.9 months (IQR: 9.8–48.5), with a maximum follow-up of 84.2 months. During the follow-up period, 30 patients died, corresponding to an overall mortality rate of 28.3%, while 76 patients remained alive at the end of the observation period.

Baseline echocardiographic characteristics of the overall population were as follows: mean LVEF was 36.9 ± 12.6%, left ventricular end-diastolic dimension (LVEDD) was 6.0 ± 0.9 cm, left ventricular end-systolic dimension (LVESD) was 4.7 ± 1.1 cm, left atrial (LA) volume was 102.3 ± 41.5 mL, and LAVI was 61.7 ± 25.6 mL/m^2^. BNP levels were elevated, with a mean value of 3230.1 ± 4401.0 pg/mL and a median of 1350 (IQR 650–3937) pg/mL. Regarding mitral regurgitation severity, 46 patients (43.4%) had grade 3 (moderate-to-severe) MR and 60 patients (56.6%) had grade 4 (severe) MR. The study population was predominantly composed of patients with secondary mitral regurgitation due to left ventricular dysfunction (83 patients, 78.3%), followed by secondary atrial mitral regurgitation (15 patients, 14.2%), while primary mitral regurgitation was less frequent (8 patients, 7.5%).

Baseline clinical characteristics, comorbidities, laboratory findings, echocardiographic parameters, and procedural complications were compared between patients according to survival status. Patients who died during follow-up were significantly older (78.13 ± 8.64 years vs. 69.46 ± 10.14 years, *p* < 0.001) and more frequently presented with advanced heart failure symptoms (Table 1). Several comorbidities, including hypertension, chronic obstructive pulmonary disease, chronic kidney disease, and prior myocardial infarction, were also more prevalent among non-survivors (Table 1).

Regarding laboratory parameters, non-survivors demonstrated significantly lower albumin and hemoglobin levels and higher bilirubin, creatinine, and brain natriuretic peptide (BNP) levels. Consequently, the ALBI score was significantly higher in the mortality group. Higher ALBI score grades were also more frequently observed among non-survivors (Table 1).

Pre-procedural TTE revealed higher left atrial (LA) volume, LAVI, pulmonary artery systolic pressure (PASP), and more severe MR in non-survivors (Table 1). Post-procedural TTE demonstrated higher PASP values, whereas trans-mitral gradients were similar between groups (Table 1). Regarding residual MR after the procedure, the mortality group had a higher rate of residual moderate-to-severe MR (grade 2–3), while mild MR (grade 1) was more common among survivors (Table 1).

Univariable Cox regression analysis was performed to identify predictors of long-term mortality. Among laboratory parameters, albumin, ALBI score, BNP, total bilirubin, hemoglobin, and creatinine were significantly associated with mortality risk (Table 2). Among echocardiographic parameters, LAVI, LA volume, pre-procedural and post-procedural PASP, tricuspid annular plane systolic excursion, higher grades of pre-procedural MR (grade 4 vs. grade 3), higher grades of post-procedural residual MR (grade 2–3 vs. grade 1), and left ventricular end-systolic and end-diastolic diameters were significantly associated with long-term mortality (Table 2). Clinical predictors included advanced age, NYHA class IV, chronic kidney disease, chronic obstructive pulmonary disease (COPD), hypertension, and prior myocardial infarction (Table 2).

**Figure 2:**
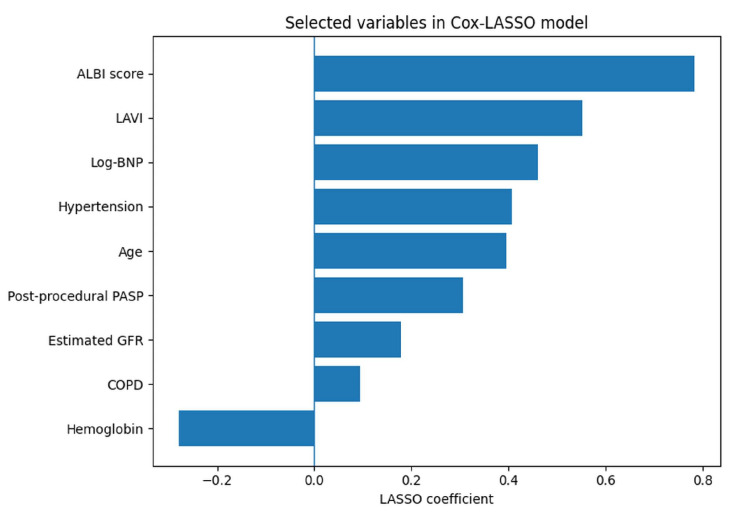
Variable selection using LASSO Cox regression. The plot illustrates the coefficients of candidate variables across different values of the regularization parameter (λ). Variables with non-zero coefficients at the optimal λ value were selected for inclusion in the multivariable model.

Variables showing significant associations in the univariable analysis were entered into a LASSO Cox regression model for variable selection. For highly correlated variables, such as LAVI and LA volume, only one of the variables was included in the LASSO model. The variables selected by the LASSO Cox regression model included ALBI score, LAVI, log-transformed BNP, hypertension, age, post-procedural PASP, estimated glomerular filtration rate (eGFR), COPD, and hemoglobin (Figure 2).

**Figure 3:**
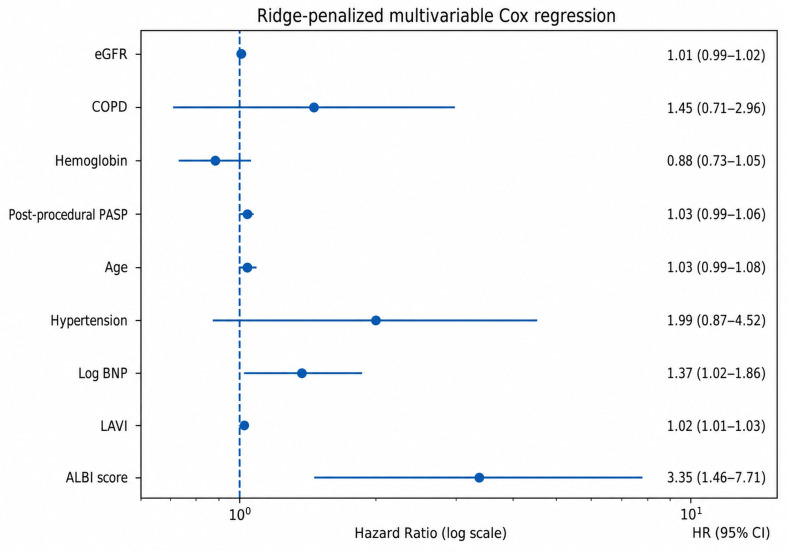
Multivariable ridge-penalized Cox regression analysis for long-term mortality. Hazard ratios (HRs) with 95% confidence intervals (CIs) are shown for variables included in the final model. The ALBI score remained independently associated with increased mortality risk.

The variables selected by the LASSO model were subsequently entered into a multivariable analysis. To reduce the risk of overfitting given the limited number of outcome events, a ridge-penalized multivariable Cox regression model was applied. In the multivariable analysis, ALBI score (HR: 3.35, 95% CI: 1.46–7.71, *p* = 0.004), LAVI (HR: 1.02, 95% CI: 1.01–1.03, *p* = 0.005), and log-transformed BNP (HR: 1.37, 95% CI: 1.02–1.86, *p* = 0.039) remained independently associated with long-term mortality (Table 3, Figure 3). Internal validation using bootstrap resampling (500 iterations) demonstrated a low degree of optimism in model performance. The apparent C-index of the ridge-penalized Cox model was 0.908, and the optimism-corrected C-index was 0.889, with a mean optimism of 0.019. The bootstrap-derived 95% confidence interval for the C-index was 0.884–0.912, indicating stable and robust discriminative ability. Pairwise correlation analysis among variables included in the multivariable model demonstrated moderate associations between several clinically related parameters. Notably, log-transformed BNP showed moderate correlations with LAVI (r = 0.50) and post-procedural pulmonary artery systolic pressure (r = 0.55), while age was moderately correlated with renal function (eGFR; r = −0.54) and hemoglobin (r = −0.54). The ALBI score also showed moderate correlations with age (r = 0.53) and log-transformed BNP (r = 0.42). Importantly, no strong correlations were observed (all correlation coefficients <0.70), indicating the absence of severe collinearity among the variables (Table S1).

In a sensitivity analysis including residual mitral regurgitation in the ridge-penalized multivariable model, residual MR was not independently associated with mortality (HR 1.16, 95% CI 0.54–2.52; *p* = 0.706). In contrast, the ALBI score remained a significant predictor of long-term mortality (HR 3.32, 95% CI 1.44–7.65; *p* = 0.005), indicating that the main findings were robust (Table S2).

To evaluate the incremental prognostic value of the ALBI score, two models were compared. Model A included age, log-transformed BNP, and LAVI, whereas Model B additionally incorporated the ALBI score. The addition of ALBI was associated with a modest increase in the model’s discriminative performance, increasing the C-index from 0.845 to 0.886 (ΔC-index = 0.041), indicating improved prediction of long-term mortality (Table 4).

At 24 months, the addition of the ALBI score to the baseline model improved risk reclassification. The continuous NRI was positive, with a bootstrap-derived 95% confidence interval ranging from 0.092 to 1.306, indicating an overall enhancement in model performance.

This improvement was primarily driven by better classification of non-events, whereas the confidence interval for event reclassification crossed zero, indicating greater uncertainty in this component.

In contrast, the event component showed greater variability, with a 95% confidence interval for the NRI events ranging from −0.120 to 0.800.

**Figure 4:**
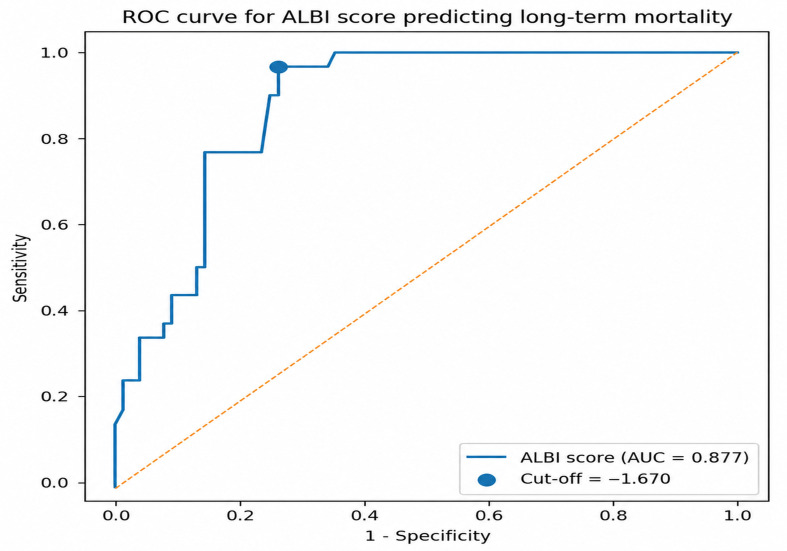
Receiver operating characteristic (ROC) curve for the ALBI score in predicting long-term mortality. The area under the curve (AUC) was 0.877, indicating good discriminative performance. The optimal cut-off value determined by Youden’s index was −1.67. The dashed diagonal line represents the line of no discrimination (AUC = 0.50), corresponding to random prediction.

ROC curve analysis was performed using a conventional approach to evaluate the ability of the ALBI score to discriminate mortality status. This analysis does not account for censoring and should therefore be interpreted with caution in the context of time-to-event data. The ALBI score demonstrated good discriminative performance, with an AUC of 0.877 (Figure 4). The optimal ALBI cut-off value determined using Youden’s index was −1.67, corresponding to a sensitivity of 96.7% and specificity of 73.7%. This threshold was subsequently used to stratify patients for survival analysis.

**Figure 5:**
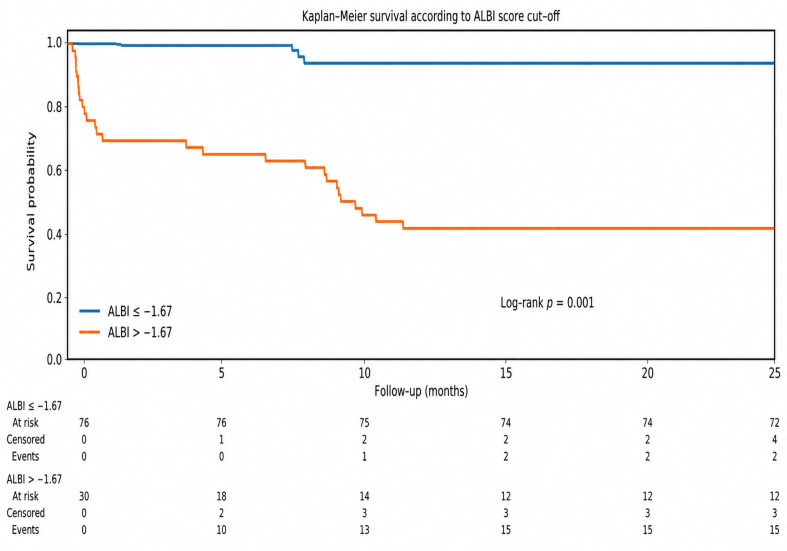
Kaplan–Meier survival curves stratified by ALBI score cut-off (−1.67).

Kaplan–Meier survival curves demonstrated lower survival probability among patients with higher ALBI scores (>−1.67) compared with those with lower ALBI scores (≤−1.67) during follow-up (Figure 5).

Patients with higher ALBI scores (>−1.67) exhibited significantly lower survival compared with those with lower ALBI scores (≤−1.67). Survival distributions between groups were compared using the log-rank test (*p* < 0.001). Follow-up was truncated at 24 months to avoid instability related to limited late observations. Numbers at risk, as well as cumulative events and censored observations, are displayed below the x-axis. Survival between groups was compared using the log-rank test, which showed a statistically significant difference between the curves (log-rank *p* < 0.001).

## 4. Discussion

In this single-center cohort of patients undergoing TEER, the baseline ALBI score was significantly associated with mortality during follow-up. Several important observations emerged from the present study. First, patients with higher baseline ALBI scores demonstrated significantly lower survival probability compared with those with lower scores. Second, the ALBI score remained independently associated with mortality after adjustment for established clinical and echocardiographic predictors. Third, the addition of the ALBI score to a clinical prediction model may provide potential incremental prognostic information, although the observed improvement in discrimination was modest and should be interpreted with caution. Finally, receiver operating characteristic analysis demonstrated good discriminatory performance of the ALBI score for mortality prediction.

Taken together, these findings suggest that congestion-related hepatic dysfunction, as reflected by the ALBI score, represents one of the important components of systemic disease burden in patients undergoing TEER. Notably, LAVI and BNP also remained independently associated with mortality, indicating that structural remodeling, neurohormonal activation, and congestion-related hepatic dysfunction may collectively contribute to adverse outcomes after TEER.

Importantly, although the present findings highlight the prognostic relevance of the ALBI score, they should be interpreted within the broader context of established post-procedural determinants after TEER. Residual mitral regurgitation, post-procedural PASP, right ventricular function, and procedural success remain key drivers of prognosis, and several of these parameters were associated with outcomes in our analyses. In this context, the relatively high rate of residual moderate-to-severe MR in our cohort should also be considered, as it may confound the relationship between systemic biomarkers and prognosis. Although residual MR was not independently associated with mortality in our sensitivity analysis, its presence may still reflect procedural and disease-related complexity and may limit the generalizability of the observed prognostic value of the ALBI score.

Accordingly, the ALBI score should be considered a complementary rather than dominant marker, reflecting the systemic and multiorgan dimension of disease burden. Its integration with established echocardiographic and procedural parameters may enhance risk stratification by capturing the impact of chronic congestion and cardio-hepatic interaction not fully reflected by cardiac measures alone.

Overall, these findings support a multidimensional approach to risk assessment after TEER, integrating cardiac, procedural, and extracardiac factors.

In the present study, the addition of the ALBI score to a clinically based model resulted in a measurable improvement in prognostic performance. Although the increase in discrimination as reflected by the C-index was modest (ΔC-index = 0.041), this degree of improvement may still be clinically meaningful, particularly in relatively homogeneous patient populations such as those undergoing TEER. Importantly, the incremental value of ALBI was further supported by net reclassification analysis. At 24 months, the inclusion of ALBI yielded a positive continuous NRI, with the bootstrap-derived confidence interval indicating an overall improvement in risk classification. Notably, this effect was primarily driven by more accurate classification of non-events, suggesting that ALBI enhances the identification of lower-risk patients. From a clinical perspective, this may help refine post-procedural risk stratification, allowing for more individualized follow-up strategies and potentially avoiding unnecessary intensive monitoring in patients with favorable profiles. While the event component of NRI showed greater variability, likely due to the limited number of events, the consistent improvement in non-event classification highlights the complementary role of ALBI beyond traditional cardiovascular parameters. However, as predefined risk categories or decision thresholds were not used, the clinical applicability of these reclassification findings remains uncertain. Taken together, these findings suggest that ALBI may provide potential incremental prognostic information in conjunction with established clinical, echocardiographic, and procedural predictors, although the observed effect appears to be primarily driven by improved classification of non-events and should be interpreted with caution. Importantly, the absence of a consistent improvement in event reclassification underscores the need for cautious interpretation of the incremental value of ALBI.

Heart failure and advanced valvular heart disease are increasingly recognized as systemic syndromes involving complex multiorgan interactions. Among these interactions, the cardio-hepatic axis plays a central role in determining clinical outcomes. Elevated right-sided cardiac filling pressures lead to hepatic venous congestion, sinusoidal dilatation, and impaired hepatic perfusion, ultimately resulting in congestive hepatopathy [13]. In patients with severe mitral regurgitation, chronic volume overload of the left atrium and pulmonary circulation may promote pulmonary hypertension and secondary right ventricular dysfunction, thereby increasing systemic venous pressures and hepatic congestion [14]. As a consequence, abnormalities such as hypoalbuminemia and hyperbilirubinemia frequently develop and may reflect advanced systemic disease.

Traditional hepatic scoring systems such as the Child–Pugh and MELD scores were originally developed for primary liver disease and may not adequately capture congestion-related hepatic dysfunction in cardiovascular populations [15]. In contrast, the ALBI score relies exclusively on albumin and bilirubin levels, providing a simple and objective measure of hepatic function. Previous studies have demonstrated that ALBI is associated with outcomes in heart failure and cardiovascular populations [6,16]. These findings further support the concept that hepatic dysfunction in heart failure reflects not only intrinsic hepatic injury but also systemic hemodynamic compromise and multiorgan venous congestion.

Increasing evidence suggests that markers of hepatic dysfunction may provide important prognostic information in patients undergoing structural heart interventions. In patients undergoing transcatheter aortic valve implantation (TAVI), impaired hepatic function assessed using MELD-XI or ALBI has been associated with worse clinical outcomes and increased mortality [7,17]. However, data regarding hepatic dysfunction in the context of TEER remain limited. Most previous studies investigating outcomes after MitraClip therapy have primarily focused on cardiac parameters such as left ventricular function, pulmonary artery pressures, residual mitral regurgitation, and procedural success rather than extracardiac organ dysfunction [10,18,19]. More contemporary registry and post-approval studies, including the EXPAND study, have further demonstrated favorable procedural success and sustained clinical improvement following TEER in real-world populations [20]. These contemporary data emphasize that outcomes after TEER are determined not only by procedural success but also by the burden of systemic disease and multiorgan dysfunction present at the time of intervention. Recent registry analyses have highlighted that extracardiac organ dysfunction, including renal and hepatic impairment, may substantially influence outcomes following transcatheter mitral valve repair [21].

The present study extends these observations by demonstrating that baseline ALBI score independently predicts mortality after TEER. These findings suggest that systemic congestion and hepatic dysfunction may represent previously underrecognized determinants of prognosis following transcatheter mitral valve repair and highlight the importance of incorporating extracardiac organ function into risk stratification models.

Given the clinical interrelationship between variables reflecting congestion, renal function, and systemic status, a certain degree of correlation among predictors is expected in this population. In our analysis, several variables demonstrated moderate pairwise correlations, particularly between BNP, echocardiographic parameters, and markers of renal function and hemoglobin, which likely reflect shared underlying pathophysiological pathways. Importantly, no strong correlations were observed, suggesting the absence of severe collinearity. To further account for potential interdependencies among predictors, we used ridge-penalized Cox regression, which is robust in the presence of correlated variables and provides stable coefficient estimates. Notably, the ALBI score remained an independent predictor despite these interrelationships, supporting its complementary prognostic value beyond conventional cardiovascular and systemic markers.

Although TEER has become an established treatment option for patients with severe mitral regurgitation who are at high surgical risk, outcomes after the procedure remain heterogeneous. Large randomized trials such as the COAPT trial and MITRA-FR trial demonstrated that treatment benefit may vary considerably depending on patient selection, ventricular remodeling, and severity of heart failure [2,3]. The baseline characteristics of our cohort were broadly comparable to major TEER trials, while also reflecting important differences in patient selection. Left ventricular dimensions and function were similar to those reported in the COAPT trial (LVEDD 6.0 vs. 6.2 cm, LVESD 4.7 vs. 5.3 cm, LVEF 36.9% vs. 31.3%), suggesting a comparable degree of ventricular remodeling, although slightly better ventricular function in our population. The severity of mitral regurgitation was also similar, with grade 3 MR present in 43.4% and grade 4 MR in 56.6% of patients, closely mirroring the distribution observed in COAPT (49.0% and 51.0%, respectively). In contrast, BNP levels were markedly higher in our cohort (median 1350 [IQR 650–3937] pg/mL; mean 3230 ± 4401 pg/mL) compared with both COAPT (1014.8 ± 1086.0 pg/mL) and MITRA-FR (median 765 [IQR 417–1281] pg/mL), suggesting a population with a higher burden of systemic congestion and more advanced disease. Importantly, while landmark trials such as COAPT and MITRA-FR predominantly enrolled patients with secondary MR due to reduced left ventricular ejection fraction, our cohort also included smaller proportions of patients with atrial functional MR and primary MR. Although the majority of our population still consisted of patients with reduced ejection fraction, the inclusion of these additional subgroups likely contributed to the slightly higher LVEF and relatively smaller ventricular dimensions observed in our study. Importantly, the inclusion of different MR etiologies, including primary, ventricular secondary, and atrial functional MR, introduces clinical heterogeneity that may influence post-TEER outcomes and should be considered when interpreting the present findings. This approach reflects a real-world clinical setting, where TEER is applied across a broader spectrum of MR etiologies. We deliberately did not exclude these subgroups in order to preserve the representativeness of the cohort. However, the sample size was not sufficient to perform adequately powered subgroup analyses. Future studies with larger populations are warranted to investigate whether the prognostic value of the ALBI score differs across specific MR subtypes, including primary, ventricular secondary, and atrial functional MR.

More recent observational studies and registry analyses have confirmed that comorbid conditions, systemic congestion, renal dysfunction, and frailty substantially influence outcomes after TEER [22,23]. Therefore, improving pre-procedural risk stratification remains an important clinical objective. Biomarkers reflecting systemic disease burden and multiorgan dysfunction may help identify patients who are most likely to benefit from transcatheter interventions. In this context, the ALBI score offers several advantages. Because albumin and bilirubin are routinely measured laboratory parameters, the ALBI score represents a simple and widely available biomarker that may complement traditional cardiac risk markers and provide additional insight into systemic congestion and cardio-hepatic interaction [24]. These findings suggest that assessment of cardio-hepatic interaction using simple laboratory markers such as the ALBI score may help identify patients with advanced systemic congestion who remain at high risk despite technically successful TEER.

## 5. Conclusions

In patients undergoing transcatheter edge-to-edge mitral repair, the baseline ALBI score is independently associated with mortality and may provide incremental prognostic value beyond conventional clinical and echocardiographic parameters. These findings highlight the importance of cardio-hepatic interaction and systemic congestion in determining outcomes after TEER. Incorporation of the ALBI score into pre-procedural risk assessment may contribute to risk stratification; however, its role in clinical decision making remains to be established. Prospective multicenter studies are warranted to validate these findings and further define the role of ALBI-based risk stratification in patients undergoing TEER.

## 6. Limitations

Several limitations of the present study should be acknowledged. First, this was a single-center study with a relatively limited sample size, which may limit the generalizability of the findings. Although penalized regression methods and bootstrap internal validation were applied to reduce overfitting, the relatively small sample size and number of events may still limit model stability. In addition, the absence of external validation warrants cautious interpretation of the predictive performance of the model. Furthermore, the ROC analysis was performed using a conventional approach that does not account for censoring; therefore, given the time-to-event nature of the primary endpoint, its results should be interpreted with caution, and time-dependent ROC methods may provide a more appropriate assessment in future studies.

Second, the retrospective design introduces the potential for selection bias and unmeasured confounding.

Third, hepatic function was assessed only using baseline laboratory measurements, and temporal changes in ALBI score during follow-up were not evaluated.

Fourth, the study population included heterogeneous mitral regurgitation phenotypes, including primary MR, ventricular secondary MR, and atrial functional MR. These subtypes differ in pathophysiology, ventricular remodeling, and prognosis after TEER, and may therefore influence post-procedural outcomes through distinct mechanisms. Due to the limited sample size, subgroup analyses were not feasible; thus, the potential differential impact of MR etiology on outcomes and on the prognostic value of ALBI could not be assessed. This heterogeneity represents an important limitation and may affect the generalizability of the findings.

Fifth, although the ALBI score was independently associated with mortality, a causal relationship between hepatic dysfunction and adverse outcomes cannot be definitively established. Prospective multicenter studies with larger patient populations are warranted to confirm these findings and further clarify the role of cardio-hepatic biomarkers in patients undergoing TEER.

Sixth, the relatively high rate of significant residual MR in our cohort may limit the interpretability of the ALBI score’s prognostic significance. While the impact of extra-cardiac biomarkers might differ in cohorts with more uniform procedural success, our sensitivity analysis confirmed that the ALBI score remained independently associated with mortality regardless of residual MR grade, suggesting its utility even in patients with suboptimal procedural outcomes.

Finally, the use of all-cause mortality as the primary endpoint may be considered a limitation, as cause-specific mortality data were not consistently available due to the retrospective design. Therefore, competing risks such as non-cardiovascular death could not be formally assessed. However, given that the ALBI score reflects systemic and multiorgan dysfunction, all-cause mortality may represent a clinically relevant and comprehensive outcome. Future prospective studies with detailed cause-of-death adjudication are warranted to better evaluate the impact of competing risks.

## Figures and Tables

**Table 1:** Comparison between survivors and non-survivors.

Variable	Alive (*n = 76*)	Dead (*n = 30*)	*p*-Value
Baseline Clinical Characteristics			
Age (years)	69.46 ± 10.14	78.13 ± 8.64	<0.001
Female sex	27 (35.5%)	8 (26.7%)	0.519
BMI (kg/m^2^)	24.54 ± 4.10	22.92 ± 2.93	0.119
SBP (mmHg)	120.13 ± 14.93	117.70 ± 8.88	0.380
DBP (mmHg)	70.43 ± 10.13	70.57 ± 9.18	0.866
NYHA III	53 (69.7%)	9 (30.0%)	<0.001
NYHA IV	23 (30.3%)	21 (70.0%)	
Comorbidities			
Diabetes mellitus	21 (27.6%)	12 (40.0%)	0.314
Hypertension	37 (48.7%)	26 (86.7%)	<0.001
CAD	63 (82.9%)	28 (93.3%)	0.223
Atrial fibrillation	31 (40.8%)	12 (40.0%)	1.000
Congestive heart failure	49 (64.5%)	24 (80.0%)	0.186
COPD	10 (13.2%)	12 (40.0%)	0.005
CKD	10 (13.2%)	12 (40.0%)	0.005
Prior myocardial infarction	27 (35.5%)	20 (66.7%)	0.007
Prior revascularization	53 (69.7%)	24 (80.0%)	0.409
Laboratory Parameters			
Albumin (g/dL)	3.30 ± 0.44	2.68 ± 0.29	<0.001
Total bilirubin (mg/dL)	1.09 ± 0.28	1.48 ± 0.60	<0.001
Hemoglobin (g/dL)	12.40 ± 1.92	10.41 ± 1.89	<0.001
WBC (×10^3^/µL)	8.93 ± 5.22	7.67 ± 2.26	0.589
Platelet (×10^3^/µL)	225.04 ± 84.18	184.97 ± 79.06	0.019
Creatinine (mg/dL)	1.36 ± 0.86	1.95 ± 1.08	<0.001
eGFR (mL/min/1.73 m^2^)	63.91 ± 26.90	42.64 ± 24.13	<0.001
CRP (mg/L)	3.40 ± 1.38	3.73 ± 1.09	0.338
BNP (pg/mL)	1100 [590–2500]	4375 [3410–9050]	<0.001
ALBI score	−1.98 ± 0.43	−1.37 ± 0.30	<0.001
ALBI grade 1	7 (9.2%)	0 (0%)	<0.001
ALBI grade 2	63 (82.9%)	17 (56.7%)	
ALBI grade 3	6 (7.9%)	13 (43.3%)	
Echocardiographic Parameters			
LVEF (%)	37.96 ± 13.40	34.17 ± 10.18	0.150
LVEDD (cm)	5.89 ± 0.88	6.25 ± 0.87	0.075
LVESD (cm)	4.56 ± 1.09	4.99 ± 1.03	0.058
LA volume (mL)	90.89 ± 36.86	131.33 ± 38.65	<0.001
LAVI (mL/m^2^)	53.86 ± 22.43	81.47 ± 22.56	<0.001
TAPSE (cm)	1.83 ± 2.37	1.39 ± 0.25	0.014
PASP (mmHg)	49.89 ± 15.10	61.13 ± 7.17	<0.001
MR subtypes			0.169
Primary MR	8 (10.5%)	0 (0.0%)	
Secondary MR (LV dysfunction)	57 (75.0%)	26 (86.7%)	
Atrial secondary MR	11 (14.5%)	4 (13.3%)	
MR grade 3	50 (65.8%)	10 (33.3%)	0.005
MR grade 4	26 (34.2%)	20 (66.7%)	
Residual transmitral gradient (mmHg)	4.68 ± 1.13	4.63 ± 1.07	0.689
Post-procedural PASP (mmHg)	43.58 ± 13.71	55.80 ± 7.53	<0.001
Residual MR grade 1	36 (47.4%)	5 (16.7%)	0.002
Residual MR grade 2	40 (52.6%)	23 (76.7%)	
Residual MR grade 3	0 (0%)	2 (6.7%)	
Procedural Complications			
Vascular complication	3 (3.9%)	2 (6.7%)	0.620
Tamponade	1 (1.3%)	1 (3.3%)	0.488
Embolic complication	3 (3.9%)	1 (3.3%)	1.000
Transfusion requirement	4 (5.3%)	3 (10.0%)	0.401

BMI: Body mass index, SBP: Systolic blood pressure, DBP: Diastolic blood pressure, NYHA: New York Heart Association, CAD: Coronary artery disease, COPD: Chronic obstructive pulmonary disease, CKD: Chronic kidney disease, WBC: White blood cell (Leukocytes), eGFR: Estimated glomerular filtration rate, CRP: C-Reactive protein, BNP: B-type natriuretic peptide, ALBI score: Albumin–bilirubin score, LVEF: Left ventricular ejection fraction, LVEDD: Left ventricular end-diastolic dimension, LVESD: Left ventricular end-systolic dimension, LA volume: Left atrial volume, LAVI: Left atrial volume index, TAPSE: Tricuspid annular plane systolic excursion, PASP: Pulmonary artery systolic pressure, and MR: Mitral regurgitation.

**Table 2:** Univariable predictors of long-term mortality.

Variable	Hazard Ratio	95% CI	*p*-Value
Albumin	0.06	0.02–0.15	<0.001
ALBI score	18.68	7.05–49.50	<0.001
Log-transformed BNP	2.57	1.80–3.67	<0.001
LAVI	1.04	1.02–1.05	<0.001
Total bilirubin	3.74	2.21–6.34	<0.001
Left atrial volume	1.02	1.01–1.03	<0.001
Age	1.10	1.06–1.16	<0.001
Hemoglobin	0.70	0.59–0.83	<0.001
Post-procedural PASP	1.06	1.03–1.10	<0.001
CKD	4.03	1.92–8.46	<0.001
NYHA IV vs. III	4.17	1.90–9.16	<0.001
eGFR	0.98	0.96–0.99	<0.001
PASP	1.05	1.02–1.09	<0.001
MR grade 4 vs. 3	3.81	1.73–8.38	<0.001
COPD	3.45	1.65–7.25	0.001
Hypertension	5.23	1.82–15.04	0.002
Prior myocardial infarction	3.31	1.51–7.27	0.003
Creatinine	1.49	1.12–1.97	0.006
Residual MR grade 2–3 vs. 1	3.64	1.39–9.54	0.009
TAPSE	0.18	0.05–0.66	0.010
LVESD	1.43	1.03–1.99	0.031
LVEDD	1.52	1.02–2.27	0.041
Congestive heart failure	2.35	0.90–6.16	0.082
Body mass index	0.91	0.82–1.01	0.086
Platelets	1.00	0.99–1.00	0.091
Coronary artery disease	5.25	0.71–38.60	0.103
LVEF	0.97	0.94–1.01	0.108
Prior revascularization	2.01	0.77–5.28	0.155
C-reactive protein	1.20	0.90–1.59	0.217
Diabetes mellitus	1.58	0.74–3.34	0.235
Leucocytes	0.94	0.84–1.05	0.253
Female sex	0.62	0.27–1.45	0.270
Transfusion requirement	1.80	0.54–5.94	0.336
Vascular complication	1.94	0.46–8.18	0.365
Systolic blood pressure	0.99	0.97–1.02	0.467
Tamponade	1.92	0.26–14.10	0.524
Residual transmitral gradient	0.97	0.67–1.39	0.860
Atrial fibrillation	1.04	0.50–2.18	0.911
Diastolic blood pressure	1.00	0.97–1.04	0.938
Embolic complication	0.93	0.13–6.81	0.940

CI: Confidence interval, ALBI score: Albumin–bilirubin score, Log-transformed BNP: Logarithmic transformed B-type natriuretic peptide, LAVI: Left atrial volume index, PASP: Pulmonary artery systolic pressure, CKD: Chronic kidney disease, NYHA: New York Heart Association, eGFR: Estimated glomerular filtration rate, MR: Mitral regurgitation, COPD: Chronic obstructive pulmonary disease, TAPSE: Tricuspid annular plane systolic excursion, LVEDD: Left ventricular end-diastolic dimension, LVESD: Left ventricular end-systolic dimension, and LVEF: Left ventricular ejection fraction.

**Table 3:** Ridge-penalized multivariable Cox regression analysis for long-term mortality.

Variable	Hazard Ratio	95% CI	*p*-Value
ALBI score	3.35	1.46–7.71	0.004
LAVI	1.02	1.01–1.03	0.005
Log-transformed BNP	1.37	1.02–1.86	0.039
Hypertension	1.99	0.87–4.52	0.101
Age	1.03	0.99–1.08	0.117
Post-procedural PASP	1.03	0.99–1.06	0.124
Hemoglobin	0.88	0.73–1.05	0.156
COPD	1.45	0.71–2.96	0.307
eGFR	1.01	0.99–1.02	0.506

CI: Confidence interval, ALBI score: Albumin–bilirubin score, LAVI: Left atrial volume index, Log- transformed BNP: Logarithmic transformed B-type natriuretic peptide, Post-procedural PASP: Post-procedural pulmonary artery systolic pressure, COPD: Chronic obstructive pulmonary disease, and eGFR: Estimated glomerular filtration rate.

**Table 4:** Incremental prognostic value of ALBI score.

Model	Variables Included	C-Index
Model A	Age, log-transformed BNP, LAVI	0.845
Model B	Age, log-transformed BNP, LAVI, ALBI score	0.886

Log-transformed BNP: Logarithmic transformed B-type natriuretic peptide, LAVI: Left atrial volume index, and ALBI score: Albumin–bilirubin score.

## Data Availability

The datasets generated and/or analyzed during the current study are not publicly available due to institutional policies and the protection of patient confidentiality. De-identified data may be made available from the corresponding author upon reasonable request, subject to approval by the institutional ethics committee.

## Supplementary Materials

The following supporting information can be downloaded at: https://www.mdpi.com/article/10.3390/medicina62050944/s1, Table S1: Correlation matrix of variables included in the multivariable model; Table S2: Sensitivity analysis including residual mitral regurgitation in the ridge-penalized multivariable Cox model.

## Abbreviations

The following abbreviations are used in this manuscript:

TEER	Transcatheter edge-to-edge repair
ALBI	Albumin–bilirubin
C-index	Concordance index
ROC	Receiver operating characteristic
LAVI	Left atrial volume index
MR	Mitral regurgitation
HF	Heart failure
CKD	Chronic kidney disease
COPD	Chronic obstructive pulmonary disease
MELD	Model for End-Stage Liver Disease
LVEF	Left ventricular ejection fraction
TTE	Transthoracic echocardiography
TEE	Transesophageal echocardiography
IQR	Interquartile range
LASSO	Least absolute shrinkage and selection operator
PASP	Pulmonary artery systolic pressure
NYHA	New York Heart Association
