# Supplementary material for: Prognostic Impact of Baseline Albumin–Bilirubin Score on Mortality After Transcatheter Edge-to-Edge Mitral Repair
Source: Medicina (Kaunas). 2026 May 12;62(5):944. doi: 10.3390/medicina62050944 (PMC13208146; doi:10.3390/medicina62050944)
Supplement: Supplementary file 1 [file medicina-62-00944-s001.zip › medicina-4243932-supplementary.pdf]

Supplementary Materials:

Table S1: Correlation matrix of variables included in the multivariable model

Variable	ALBI score	Age	Log BNP	LAVI	Post-procedural PASP	eGFR	Hemoglobin	Hypertension	COPD
ALBI score	1.00	0.53	0.42	0.36	0.39	-0.33	-0.36	0.31	0.29
Age	0.53	1.00	0.32	0.20	0.23	-0.54	-0.54	0.59	0.42
Log-transformed BNP	0.42	0.32	1.00	0.50	0.55	-0.30	-0.24	0.22	0.24
LAVI	0.36	0.20	0.50	1.00	0.48	-0.28	-0.28	0.00	0.18
Post-procedural PASP	0.39	0.23	0.55	0.48	1.00	-0.26	-0.31	0.08	0.11
eGFR	-0.33	-0.54	-0.30	-0.28	-0.26	1.00	0.55	-0.57	-0.29
Hemoglobin	-0.36	-0.54	-0.24	-0.28	-0.31	0.55	1.00	-0.57	-0.23
Hypertension	0.31	0.59	0.22	0.00	0.08	-0.57	-0.57	1.00	0.33
COPD	0.29	0.42	0.24	0.18	0.11	-0.29	-0.23	0.33	1.00

ALBI score: Albumin-bilirubin score, Log- transformed BNP: Logarithmic transformed b-type natriuretic peptide, LAVI: Left atrial volume index, Post-procedural PASP: Post-procedural pulmonary artery systolic pressure, eGFR: Estimated glomerular filtration rate, COPD: Chronic obstructive pulmonary disease.

Table S2: Sensitivity analysis including residual mitral regurgitation in the ridge-penalized multivariable Cox model.

Variable	Hazard Ratio (95% CI)	p-value
ALBI score	3.32 (1.44–7.65)	0.005
LAVI (per 1 mL/m^2 increase)	1.02 (1.01–1.03)	0.006
Log-transformed BNP	1.37 (1.02–1.85)	0.043
Hypertension	1.96 (0.86–4.47)	0.110
Age (per year)	1.03 (0.99–1.08)	0.115
Post-procedural PASP (per mmHg)	1.02 (0.99–1.06)	0.137
Hemoglobin (per g/dL)	0.88 (0.73–1.05)	0.158
COPD	1.46 (0.71–2.99)	0.298
eGFR (per mL/min/1.73 m^2)	1.01 (0.99–1.02)	0.509
Residual MR (grade 2–3 vs grade 1)	1.16 (0.54–2.52)	0.706

CI: Confidence interval, ALBI score: Albumin-bilirubin score, LAVI: Left atrial volume index, Log- transformed BNP: Logarithmic transformed b-type natriuretic peptide, Post-procedural PASP: Post-procedural pulmonary artery systolic pressure, COPD: Chronic obstructive pulmonary disease, eGFR: Estimated glomerular filtration rate, MR: Mitral regurgitation.
